# Volumetry of Selected Brain Regions—Can We Compare MRI Examinations of Different Manufacturers and Field Strengths?

**DOI:** 10.1007/s00062-024-01489-x

**Published:** 2025-01-20

**Authors:** Svea Seehafer, Lars-Patrick Schmill, Sönke Peters, Olav Jansen, Schekeb Aludin

**Affiliations:** Department of Radiology and Neuroradiology, University Hospital of Schleswig-Holstein, Arnold-Heller-Str. 3, Hs D (Neurozentrum), 24105 Kiel, Germany

**Keywords:** Magnetic Resonance Imaging, MRI Hardware, Neuroimaging, Brain Volumetry, Automatic Brain Segmentation

## Abstract

**Purpose:**

Magnetic Resonance Imaging based brain segmentation and volumetry has become an important tool in clinical routine and research. However the impact of the used hardware is only barely investigated. This study aims to assess the influence of scanner manufacturer, field strength and head-coil on volumetry results.

**Methods:**

10 healthy subjects (27.4 ± 1.71 years) were prospectively examined in a Philips Achieva 1.5T, Philips Ingenia CX 3T, Siemens MAGNETOM Aera 1.5T and Siemens MAGNETOM Vida 3T, the latter equipped with three different head coils, within one day. Brain volumetry of the whole brain, total white and grey matter, the cortical grey matter of the supratentorial lobes as well as regions important for the differentiation of neurodegenerative diseases of the dementia and movement disorder spectrum and the ventricular system was performed using the CE-certified software mdbrain by mediaire (Berlin, Germany). Both raw volumetry results and percentile allocation provided by the software were analysed.

**Results:**

This study reveals significantly different volumetry results for all examined brain regions beside the ventricular system between the different MRI devices but comparable results between the different head coils. When examining the percentile allocation provided by used software, the Intraclass-Correlation-Coefficient (ICC) values were even lower than the raw volume ICC values ranging from poor to excellent correlation.

**Conclusion:**

The present study reveals highly relevant results that need to be considered both in clinical routine when analysing follow-up examinations from different scanner types and clinical research, especially when planning longitudinal and/or multicentre studies.

## Introduction

**Fig. 1:**
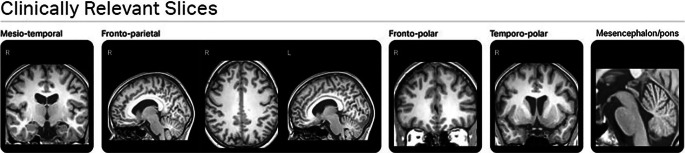
Clinically relevant slices derived from mdbrain. Seven different slices of a 3D-T1 image divided into five groups. These slices are important for visual evaluation of brain atrophy signs

Magnetic Resonance Imaging (MRI) based brain segmentation and volumetry has become an important tool in clinical routine. It is increasingly established as a powerful tool in the diagnosis, understanding and monitoring of especially neurodegenerative diseases [1] namely dementia syndromes such as Alzheimer’s disease or movement disorders such as Parkinon’s disease to name the most common ones. For long time, the assessment of brain volume was based on visual and qualitative parameters such as for example the Global Cortical Atrophy Scale [2], the Medial Temporal Lobe Atrophy Scale [3], the Koedam Score for parietal atrophy ([4]; Fig. 1) or the Fazekas Scale for assessment of White Matter Hyperintensities [5]. A task that is highly subjective.

In recent years, due to technical development, three dimensional MRI sequences based on isometric voxels have become widely available. Because of acquisition of thin slices without interslice gap, they offer the possibility of multiplanar reconstruction. Concomitant with this, an increasing number of semi- or fully automated tools for brain segmentation and volumetry are being developed. Currently, application of such products increases in clinical routine, e.g. for brain volumetry, evaluation of atrophy pattern, as well as quantitative and qualitative assessment of potential lesions. Some programs also provide a percentile classification of the obtained results. As part of these developments, the number of studies using tools for automated brain volumetry constantly expands. However, research centres and hospitals nowadays are equipped with varying MRI scanners with different field strengths and from diverse manufacturers provided with different receiver coils. This heterogeneity of hardware may cause a potential bias and thus reduced comparability of studies and clinical examinations. Some studies already investigated the influence of segmentation tools on volumetry results [6, 7] but only few studies focused on the impact of the used hardware [8]. This study therefore aims to assess the influence of scanner manufacturer, field strength and acquisition coil on the results of fully automated brain volumetry using the commercially available CE-certified software mdbrain from mediare (mediare, Berlin, Germany). Results could have a major impact on the comparability of study results and the reliability of clinical evaluation of atrophy progression. Accordingly, for planning large multicentre and/or longitudinal studies, these results need to be considered. They might have important consequences for both diagnostic and therapeutic decisions.

## Materials and Methods

### Subjects

In this prospective study ten healthy subjects were included. Exclusion criteria consisted of electric implants such as defibrillators, neurostimulators, pacemakers and other standard MRI contraindications. Other exclusion criteria were brain tumours and other neurological disorders that could cause cognitive impairment. Also neurosurgical procedures with influence on the integrity of the brain parenchyma in subject’s history were exclusion criteria. All subjects provided written consent in the study. This work was performed in accordance with the ethical standards laid down in the 1964 Declaration of Helsinki and its later amendments. The study was approved by the local ethical committee.

#### MRI Acquisition

For this prospective study, four different MRI systems were used. In detail these were Philips Achieva 1.5 Tesla (T) (Philips Healthcare, Best, The Netherlands) equipped with a 8-channel receiver head coil, Philips Ingenia CX 3T (Philips Healthcare, Best, The Netherlands) equipped with a 32-channel receiver head coil, Siemens MAGNETOM Aera 1.5T (Siemens Healthcare, Erlangen, Germany) equipped with 20-channel head receiver coil and Siemens MAGNETOM Vida 3T (Siemens Healthcare, Erlangen, Germany) equipped with a 20-, 32- and 64-channel head receiver coil. All scanners were located on the same clinic campus. All subjects were examined parallel in the same timeslot at the same day at all four scanners in a randomized manner and with all three receiver coils for the MAGNETOM Vida 3T. Total study time did not exceed four hours per subject. Scan parameters of the different scanners are listed in Table 1 and were kept constant for all individuals and coils.

**Table 1:** Imaging parameters

	Philips Achieva 1.5T	Philips Ingenia CX 3T	MAGNETOM Aera 1.5T	MAGNETOM Vida 3T
Sequence name	T1-TFE	T1-TFE	T1-MPRAGE	T1-MPRAGE
Echo time (ms)	3.2 (shortest)	3 (shortest)	2.68	1.91
Repetition time (ms)	7.1 (shortest)	6.6 (shortest)	1890	2000
Inversion time (ms)	Min. 855.08	Min. 587.15	1110	920
Flip angle (°)	8	8	8	9
Voxel size (mm)	1 × 1 × 1	1 × 1 × 1	1 × 1 × 1	0.8 × 0.8 × 0.9
Acquisition plane	Sagittal	Sagittal	Sagittal	Sagittal
Acquisition time (min:sec)	5:59	3:11	4:02	4:51

### Image Analysis

**Fig. 2:**
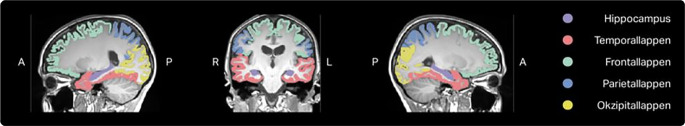
Example of automated segmentation performed by mdbrain. The volumetric results of the different brain areas are provided by the software in a table (not shown)

**Fig. 3:**
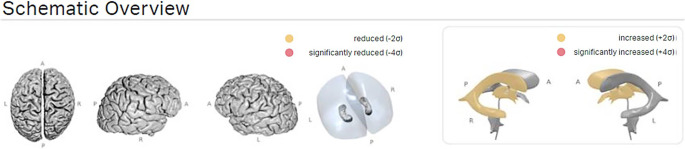
Example of schematic overview of the brain and ventricular system from different angles. In case of aberration from the normal values and percentiles, areas are coloured in yellow (more than two standard deviations) or red (more than four standard deviations)

A visual assessment of all images acquired was performed to exclude possible causes of inaccurate volumetry measurement such as motion artefacts or metal artefacts. All obtained T1-weigehted-scans were post-processed using the CE-certified Software mdbrain (mediaire, Berlin, Germany, version 4.9.1) (see Figs. 1, 2 and 3). As per the handbook provided by the company, the algorithm uses a DeepLearning based model with U‑NET-architecture. It was trained with ~3000 datasets from different scanners from Philips, Siemens and GE with different field strengths. Different augmentation techniques for contrast, resolution, rotation and elastic distortion were used to augment robustness. For the definition of the percentiles, ~8000 data were included from healthy controls aged between 10 and 99 years. Regional volumetry can be performed for the right and left side of the supratentorial lobes, some mesiotemporal areas, namely the hippocampus the parahippocampal gyrus and the entorhinal cortex, basal ganglia and thalamus as well as for infratentorial brainstem, mesencephalon, pons and cerebellar cortex and the ventricular system, split into the right and left ventricle, the third and fourth ventricle. The volumetry results were visually checked for plausibility. Furthermore, mdbrain also provides a designation of the volumetry values on percentiles under consideration of age, gender and total intracranial volume. Doing so, the program uses two cut-off values to identify pathological values: a classical definition with two standard deviations around the mean of a Gaussian distribution and a conservative definition with four standard deviations around the mean of a Gaussian curve. Values outside these ranges are either marked in yellow or red in the report (Fig. 3).

### Statistical Analysis

Normal distribution for the volumetry results was tested by the D’Agostino & Pearson test. The resulting data were statistically analysed using repeated measures one way ANOVA with Geisser-Greenhouse epsilon correction for normal distributed data and Friedmann test for not normal distributed data. Post-hoc analysis was performed between Philips Achieva 1.5T and Philips Ingenia CX 3T, Philips Achieva 1.5T and Siemens MANGETOM Aera 1.5T, Philips Ingenia 3T and Siemens MAGNETOM Vida 3T and Siemens MAGNETOM Aera 1.5T and Siemens MAGNETOM Vida 3T using Bonferroni multiple comparisons test for normal distributed data and Dunn’s multiple comparisons test for not normal distributed data. In case of Siemens MAGNETOM Vida 3T the 20-channel receiver coil was used for post-hoc comparison with Siemens MAGNETOM Aera 1.5T and the 32-channel receiver coil was used for post-hoc comparison with Philips Ingenia CX 3T.

Moreover, also the different receiver coils for Siemens MAGNETOM Vida 3T were compared the same way for normal and not normal distributed data respectively. For statistical analysis GraphPad Prism Version 10.1.2 was used.

Furthermore, as a measurement of agreement, the intraclass correlation coefficient (ICC) was calculated for both the volumetric and percentile values. The chosen model was based on two way random effects, single-score and absolute agreement using the following formula:

$$ICC=\frac{MS_{R}-MS_{E}}{MS_{R}+\left(k-1\right)*MS_{E}+\frac{k}{n}*\left(MS_{C}-MS_{E}\right)}$$

with *n* being the number of examined objects, k the number of ratings, MS_R_ the mean square rows, MS_E_ the mean square error and MS_C_ the mean square columns. Having an index ranging between 0 (no agreement) and 1 (absolute agreement), the obtained ICC values were interpreted as poor agreement for values < 0.5, as moderate agreement for values between 0.5 and 0.74, as good agreement for values between 0.75 and 0.9 and as excellent agreement for values of > 0.9 [9]. First data from all scanners were analysed followed by a pairwise comparison. Also the results of the different receiver coils from the Siemens MAGNETOM Vida 3T were compared in a separate measurement.

## Results

### Subject Demographics

Five (50%) of the 10 subjects included in this study were male and five (50%) were female. The mean patient age was 27.4 ± 1.71 years (see Table 2).

**Table 2:** Subject demographics

Inclusions (*n*)	10
Gender (m/f)	5/5 (50%/50%)
Age in years (mean ± standard deviation)	m: 27.6 ± 2.06 w: 27.2 ± 0.98

### Measurements

Automated volumetric measurements and percentile allocation computed by mdbrain software were determined for each of the following brain structures for each MRI scanner and each head coil used: whole brain, total white matter, total grey matter, cerebral cortex, brainstem, cerebellar cortex, third ventricle and fourth ventricle. For following structures measurements were computed for right and left parts: cortical volume of frontal lobe, parietal lobe, occipital lobe, temporal lobe, hippocampus, thalamus, lateral ventricle. Table 3 illustrates the results from different MRI-scanners for the before mentioned regions.

**Table 3:** Overview of means (ml), standard deviation (ml) and coefficient of variance (%) for volumetric results for the different MRI-scanners and different receiver coils in case of Siemens MAGNETOM Vida 3T. *Italicized* results represent the highest mean values

	Philips Achieva 1.5T			Philips Ingenia CX 3T			Siemens MAGNETOM Aera 1.5T			Siemens MAGNETOM Vida 3T			Sienemens MAGNETOM Vida 3T			Siemens MAGNETOM Vida 3T		
										20-channel-coil			32-channel-coil			64-channel-coil		
	Mean (ml)	Standard deviation (ml)	Coefficient of variance (%)	Mean (ml)	Standard deviation (ml)	Coefficient of variance (%)	Mean (ml)	Standard deviation (ml)	Coefficient of variance (%)	Mean (ml)	Standard deviation (ml)	Coefficient of variance (%)	Mean (ml)	Standard deviation (ml)	Coefficient of variance (%)	Mean (ml)	Standard deviation (ml)	Coefficient of variance (%)
Whole Brain	1215	120.2	9.892	1233	122.9	9.966	*1313*	133.5	10.17	1302	127.6	9.802	1302	129.7	9.96	1301	125	9.611
Total White Matter	520.2	63.55	12.22	522.2	63.75	12.21	*573.8*	68.33	11.91	530.5	61.94	11.68	532.7	62.99	11.82	521.6	65.14	12.49
Total Grey Matter	694.7	61.64	8.874	711.2	64.26	9.035	739.5	70.22	9.496	*771.3*	69.97	9.072	769.5	71	9.228	770.1	68.3	8.87
Cerebral Cortex	480.9	42.08	8.75	492.6	43.99	8.931	511.8	49.15	9,603	*536*	48.18	8.989	535	48.72	9.106	535	46.58	8.707
Frontal Lobe right	89.55	8.485	9.475	92.34	8.316	9.005	95.9	10.07	10.5	96.57	9.287	9.617	*97.04*	9.533	9.823	96.75	8.755	9.049
Frontal Lobe left	88.13	8.574	9.729	89.81	8.51	9.475	93.39	9.582	10.26	95.24	9.402	9.872	*96.02*	9.484	9.877	95.57	9.049	9.469
Parietal Lobe right	48.52	4.564	9.406	50	4.727	9.543	51.07	5.376	10.53	55.18	5.345	9.686	54.93	5.332	9.707	*55.2*	5.073	9.19
Parietal Lobe left	48.8	4.226	8.654	50.58	4.56	9.016	51.06	5.453	10.68	*55.49*	5.22	9.406	55.23	5.422	9.818	55.45	4.964	8.953
Occipital Lobe right	34.53	4.058	11.83	35.6	4.22	11.85	35.58	4.102	11.53	39.28	4.57	11.63	*39.29*	4.61	11.73	39.01	4.609	11.82
Occipital Lobe left	37.81	3.908	10.34	38.73	3.996	10.32	38.13	4.183	10.97	*42.57*	4.291	10.08	41.91	4.428	10.57	42.3	4.502	1.424
Temporal Lobe right	69.48	6.59	9.485	69.64	6.847	9.832	74.99	7.569	10.09	*77.93*	7.348	9.429	77.09	7.399	9.598	77.3	7.196	9.309
Temporal Lobe left	65.04	6.308	9.698	66.89	6.534	9.768	71.68	7.059	9.847	*73.74*	7.069	9.587	73.5	7.082	9.635	73.35	7.008	9.555
Hippocampus right	3.93	0.4001	10.18	4.04	0.4033	9.983	4.26	0.4812	11.3	*4.31*	0.4149	9.626	4.29	0.4508	10.51	4.29	0.4095	9.545
Hippocampus left	3.49	0.3281	9.402	3.61	0.3247	8.995	3.75	0.344	9.173	3.78	0.3542	9.345	*3.79*	0.3542	9.345	*3.79*	0.3348	8.835
Parahippocampal Gyrus right	3.13	0.386	12.33	3.23	0.4347	13.46	3.43	0.4473	13.04	3.52	0.4417	12.55	*3.56*	0.4904	13.77	3.54	0.4427	12.51
Parahippocampal Gyrus left	3.26	0.3806	11.68	3.43	0.3433	10.01	3.61	0.3479	9.636	3.68	0.3393	9.22	*3.75*	0.3979	10.61	3.7	0.3682	9.951
Entorhinal Cortex right	2.25	0.2953	13.13	2.31	0.3635	15.73	2.52	0.3824	15.17	2.63	0.3592	13.66	2.62	0.4211	16.07	*2.66*	0.4351	16.36
Entorhinal Cortex left	2.25	0.3206	14.25	2.37	0.3199	13.5	2.57	0.3335	12.98	*2.71*	0.3755	13.86	2.65	0.3808	14.37	2.67	0.3773	14.13
Putamen right	4.81	0.6008	12.49	4.93	0.6701	13.59	4.91	0.6244	12.72	*4.94*	0.6346	12.85	4.92	0.6494	13.2	4.9	0.6429	13.12
Putamen left	4.97	0.5794	11.66	*5.07*	0.5794	11.43	4.98	0.5884	11.82	5.03	0.587	11.67	5.04	0.631	12.52	5.03	0.587	11.67
Thalamus right	8.55	0.7892	9.23	8.65	0.7352	8.5	8.79	0.7608	8.655	*8.97*	0.8056	8.981	8.96	0.8099	9.039	8.95	0.8003	8.942
Thalamus left	8.72	0.8766	10.05	8.83	0.8525	9.655	8.96	0.8959	9.999	*9.14*	0.8758	9.583	9.08	0.8991	9.902	9.12	0.8829	9.681
Brainstem	26.64	2.827	10.61	27.12	2.889	10.65	27.69	2.913	10.52	*27.75*	2.996	10.8	27.55	2.922	10.6	27.69	3.041	10.98
Mesencephalon	7.78	0.8053	10.35	7.91	0.8452	10.68	8	0.8769	10.96	8.11	0.8582	10.58	8.05	0.8502	10.56	*8.13*	0.8381	10.31
Pons	14.38	1.671	11.62	14.58	1.673	11.47	14.83	1.723	11.62	*14.86*	1.759	11.84	14.8	1.728	11.68	14.84	1.761	11.87
Cerebellar Cortex	106.1	11.9	11.21	108.5	12.01	11.06	115.2	13.1	11.37	*120.5*	13.9	11.54	119.9	13.69	11.41	120.4	14.39	11.96
Lateral Ventricle right	6.57	3.617	55.06	6.57	3.722	56.65	6.53	3.669	56.18	*6.58*	3.706	56.32	6.56	3.616	55.12	*6.58*	3.665	55.71
Lateral Ventricle left	7.98	3.338	41.83	7.92	3.442	43.46	8.04	3.416	42.49	*8.09*	3.427	42.36	8.04	3.367	41.88	8.06	3.407	42.27
Third Ventricle	*0.74*	0.2675	36.15	0.73	0.2627	35.98	0.73	0.2627	35.98	0.71	0.2644	37.23	0.73	0.2627	35.98	0.73	0.2627	35.98
Fourth Ventricle	*1.45*	0.3894	26.86	1.4	0.383	27.36	1.42	0.405	28.52	1.41	0.4122	29.23	1.4	0.411	29.35	1.38	0.4211	30.52

Table 4 illustrates the results of the repeated measures one way ANOVA or Friedmann Test.

**Table 4:** Results of repeated measures one way ANOVA/Friedmann test for the examined brain regions. **Bold** results represent statistical significance

Brain Region	Statstic test	Result
Whole brain	Repeated measures one way ANOVA	F (1.775, 15.98) = 427.3 ***p*** **<** **0.0001**
Total white matter	Repeated measures one way ANOVA	F (1.46, 13.14) = 336.9 ***p*** **<** **0.0001**
Total grey matter	Repeated measures one way ANOVA	F (1.489, 13.40) = 395.8 ***p*** **<** **0.0001**
Cerebral cortex	Repeated measures one way ANOVA	F (1.573, 14.16) = 370.4 ***p*** **<** **0.0001**
Frontal lobe right	Repeated measures one way ANOVA	F (1.522, 13.70) = 36.88 ***p*** **<** **0.0001**
Frontal lobe left	Repeated measures one way ANOVA	F (1.485, 13.36) = 46.33 ***p*** **<** **0.0001**
Parietal lobe right	Repeated measures one way ANOVA	F (2.745, 24.70) = 178.5 ***p*** **<** **0.0001**
Parietal lobe left	Repeated measures one way ANOVA	F (2.093, 18.84) = 199.6 ***p*** **<** **0.0001**
Occipital lobe right	Repeated measures one way ANOVA	F (2.262, 20.36) = 175.3 ***p*** **<** **0.0001**
Occipital lobe left	Repeated measures one way ANOVA	F (3.128, 28.15) = 148.9 ***p*** **<** **0.0001**
Temporal lobe right	Repeated measures one way ANOVA	F (1.734, 15.60) = 248.1 ***p*** **<** **0.0001**
Temporal lobe left	Repeated measures one way ANOVA	F (2.007, 18.06) = 371.7 ***p*** **<** **0.0001**
Hippocampus right	Repeated measures one way ANOVA	F (2.933, 26.39) = 91.89 ***p*** **<** **0.0001**
Hippocampus left	Repeated measures one way ANOVA	F (2.264, 20.38) = 52.63 ***p*** **<** **0.0001**
Parahippocampal Gyrus right	Repeated measures one way ANOVA	F (2.795, 25.15) = 63.83 ***p*** **<** **0.0001**
Parahippocampal Gyrus left	Repeated measures one way ANOVA	F (3.028, 27.25) = 85.46 ***p*** **<** **0.0001**
Entorhinal Cortex right	Repeated measures one way ANOVA	F (2.934, 21.55) = 44.85 ***p*** **<** **0.0001**
Entorhinal Cortex left	Repeated measures one way ANOVA	F (2.717, 24.45) = 87.82 ***p*** **<** **0.0001**
Putamen right	Repeated measures one way ANOVA	F (3.828, 34.46) = 6.763 ***p*** **=** **0.0005**
Putamen left	Repeated measures one way ANOVA	F (2.079, 18.71) = 7.654 ***p*** **=** **0.0034**
Thalamus right	Repeated measures one way ANOVA	F (2.974, 26.77) = 78.25 ***p*** **<** **0.0001**
Thalamus left	Repeated measures one way ANOVA	F (3.188, 28.69) = 82.13 ***p*** **<** **0.0001**
Brainstem	Repeated measures one way ANOVA	F (2.549, 22.94) = 65.68 ***p*** **<** **0.0001**
Mesencephalon	Repeated measures one way ANOVA	F (1.38, 12.42) = 10.09 ***p*** **=** **0.0047**
Pons	Repeated measures one way ANOVA	F (2.687, 24.18) = 57.64 ***p*** **<** **0.0001**
Cerebellar cortex	Repeated measures one way ANOVA	F (1.187, 10.69) = 215.3 ***p*** **<** **0.0001**
Lateral ventricle right	Friedmann Test	*p* = 0.4134
Lateral ventricle left	Friedmann Test	***p*** **=** **0.0016**
Third ventricle	Repeated measures one way ANOVA	F (1.848, 16.64) = 2.157 *p* = 0.1494
Fourth ventricle	Friedmann Test	***p*** **=** **0.0302**

**Fig. 4:**
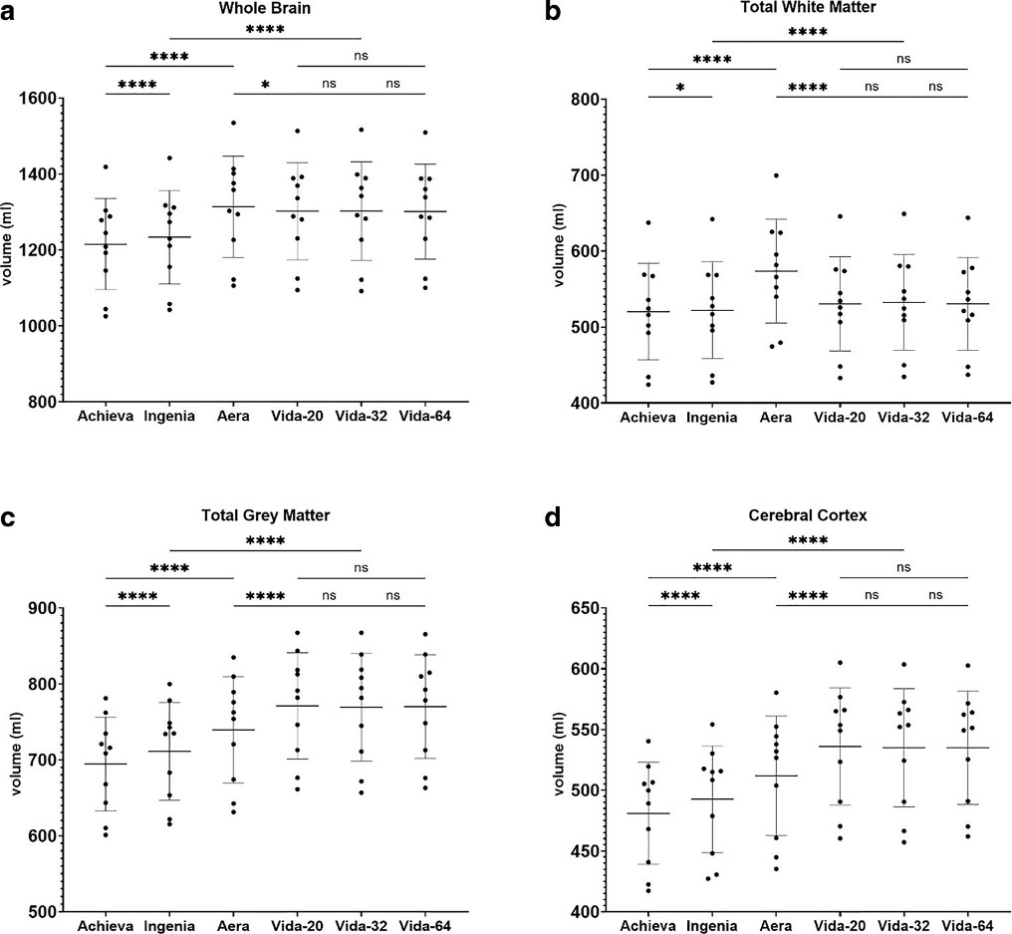
Results of post-hoc analysis between the different MRI-scanners. Vida-20, Vida-32 Fig. 2: Results of post-hoc analysis between the different MRI-scanners for **a** whole brain volume, **b** total white matter volume, **c** total grey matter volume and **d** volume of the cerebral cortex. Vida-20, Vida-32 and Vida-64 represent the results of Siemens MAGNETOM Vida 3T with the 20-, 32- or 64-channel receiver coil respectively.*ns* not significant. *: *p* < 0.05; **: *p* < 0.005; ***: *p* < 0.0005; ****: *p* < 0.0001

**Fig. 5:**
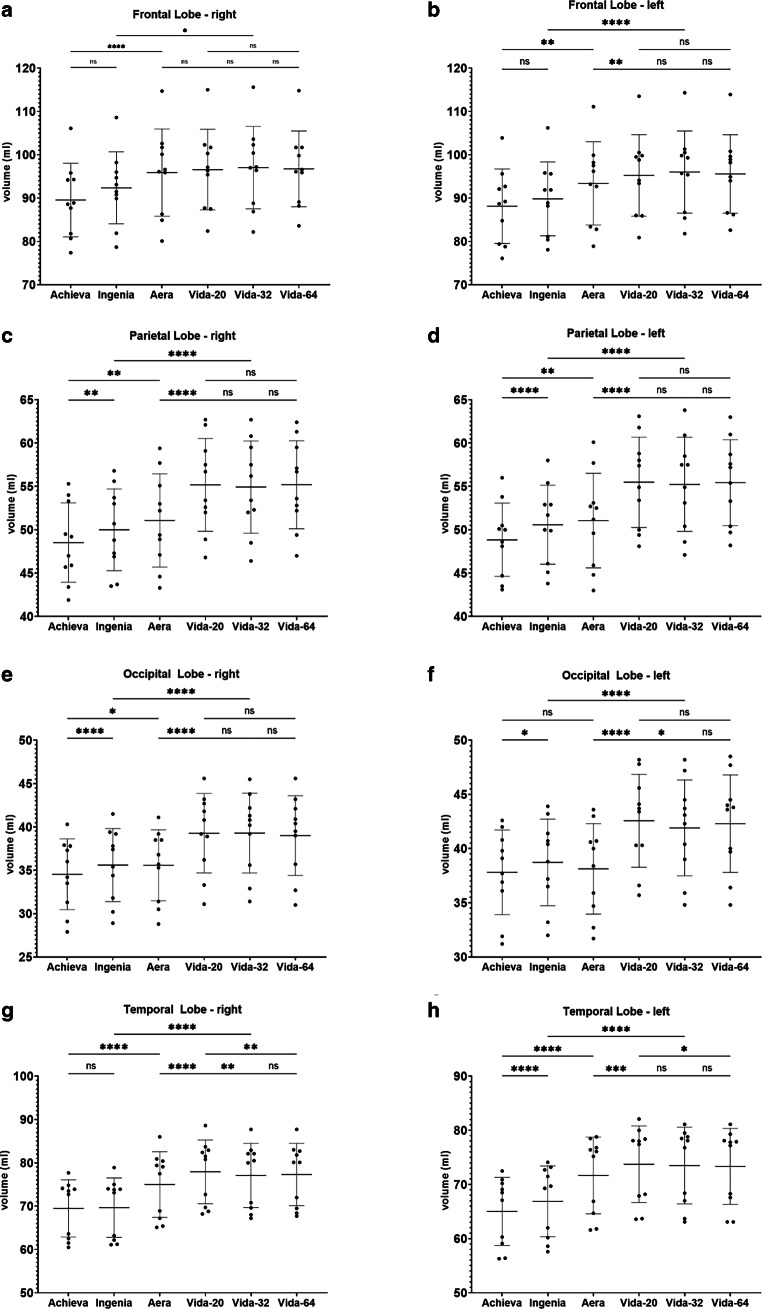
Results of post-hoc analysis between the different MRI-scanners. Vida-20, Vida-32 Fig. 2: Results of post-hoc analysis between the different MRI-scanners for **a** the right frontal lobe, **b** the left frontal lobe, **c** the right parietal lobe, **d** the left parietal lobe, **e** the right occipital lobe, **f** the left occipital lobe, **g** the right temporal lobe and **h** the left temporal lobe. Vida-20, Vida-32 and Vida-64 represent the results of Siemens MAGNETOM Vida 3T with the 20-, 32- or 64-channel receiver coil respectively. *ns* not significant. *: *p* < 0.05; **: *p* < 0.005; ***: *p* < 0.0005; ****: *p* < 0.0001

**Fig. 6:**
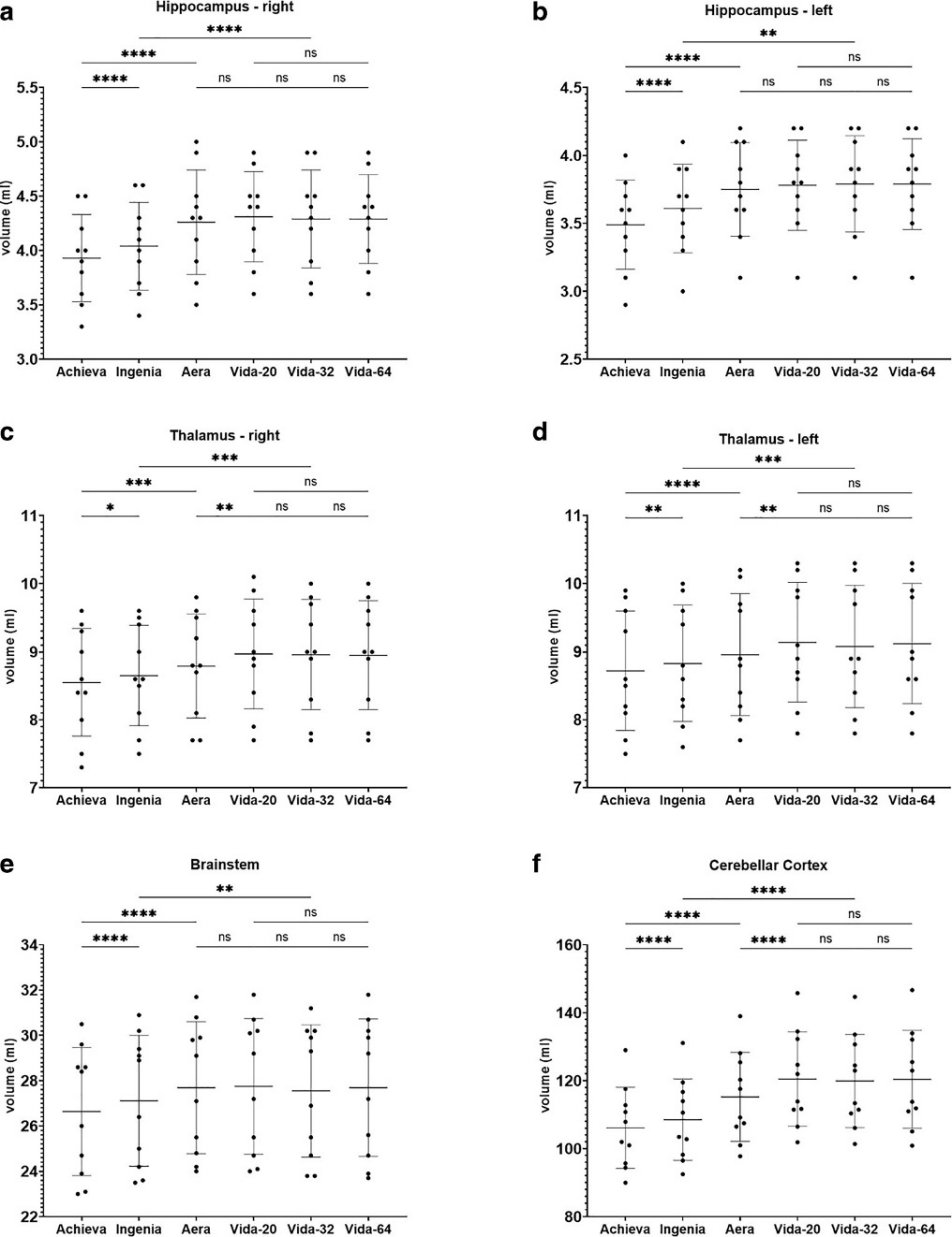
Results of post-hoc analysis between the different MRI-scanners. Vida-20, Vida-32 Fig. 2: Results of post-hoc analysis between the different MRI-scanners for **a** the right hippocampus, **b** the left hippocampus, **c** the right parahippocampal gyrus, **d** the left parahippocampal gyrus, **e** the right entorhinal cortex and **f** the left entorhinal cortex. Vida-20, Vida-32 and Vida-64 represent the results of Siemens MAGNETOM Vida 3T with the 20-, 32- or 64-channel receiver coil respectively. *ns* not significant. *: *p* < 0.05; **: *p* < 0.005; ***: *p* < 0.0005; ****: *p* < 0.0001

**Fig. 7:**
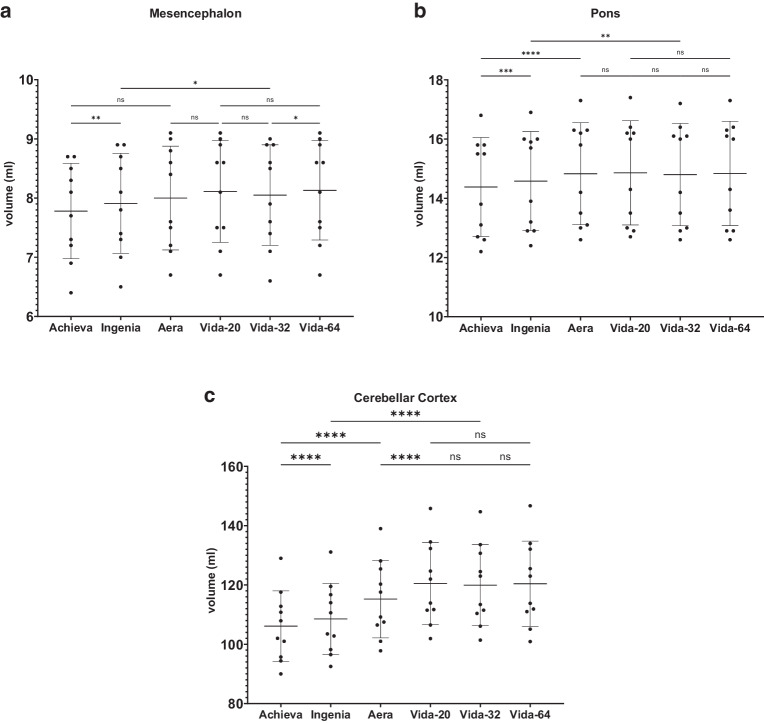
Results of post-hoc analysis between the different MRI-scanners. Vida-20, Vida-32 Fig. 2: Results of post-hoc analysis between the different MRI-scanners for **a** the mesencephalon, **b** the pons, **c** the cerebellar cortex. Vida-20, Vida-32 and Vida-64 represent the results of Siemens MAGNETOM Vida 3T with the 20-, 32- or 64-channel receiver coil respectively. *ns* not significant. *: *p* < 0.05; **: *p* < 0.005; ***: *p* < 0.0005; ****: *p* < 0.0001

**Fig. 8:**
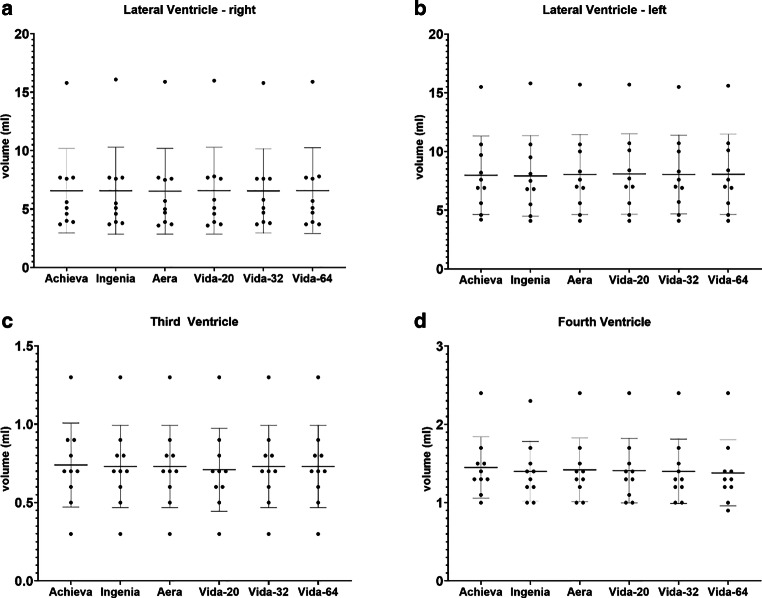
Results of post-hoc analysis between the different MRI-scanners for **a** the right lateral ventricle, **b** the left lateral ventricle, **c** the third ventricle and **d** the fourth ventricle. Vida-20, Vida-32 and Vida-64 represent the results of Siemens MAGNETOM Vida 3T with the 20-, 32- or 64-channel receiver coil respectively. No comparison bars are displayed as none of the post-hoc analyses yielded a statistic significant result

Figure 4, 5, 6, 7 and 8 illustrate the results of the post-hoc analysis between the different scanners and receiver coils. The analysis reveals significant differences in all brain regions between the applied field strengths and scanner manufacturers. The majority of differences that just missed the significance level are observed between the two Philips devices (right and left frontal lobe, right temporal lobe) as well as between the two Siemens scanners of different field strength (right and left hippocampus, brainstem). Furthermore, only a slight difference is noted between the two 1.5T devices (left occipital lobe). Regarding the ventricular system, no statistically significant differences are observed in post-hoc analysis neither between the utilized field strengths and scanner manufacturers nor among different coils.

The most highly significant differences with a *p* < 0.0001 are identified between the two 3T devices of the different manufacturers. In almost all examined regions, the measured volume on the Philips Achieva 1.5T is the smallest, while on the Siemens MAGNETOM Vida 3T it is the largest, suggesting the following pattern for volume results: Philips Achieva 1.5T < Philips Ingenia CX 3T < Siemens MAGNETOM Aera 1.5T < Siemens MAGNETOM Vida 3T. Exceptions to this trend include the total volume, where the results of Siemens MAGNETOM Aera 1.5T showed the highest values, the white matter, where also the volumetric results from Siemens MAGNETOM Aera 1.5T are the highest and the occipital lobes where volumetry results from Philips Ingenia CX 3T were slightly higher than from Siemens MAGNETOM Aera 1.5T.

In general, there are hardly any statistically significant differences between the utilized coils on the Siemens MAGNETOM Vida 3T, except for the left occipital lobe (20- vs. 32-channel coil: *p* = 0.048), the right temporal lobe (20 vs. 32-channel coil: *p* = 0.0066; 20- vs. 64-channel coil: *p* = 0.0022) and the left temporal lobe (20- vs. 64-channel coil: 0.0484).

Table 5 illustrates the results of the ICC for the analysis of all used scanners and receiver coils as well as the pairwise analysis in the line of the post-hoc analyses mentioned above.

**Table 5:** ICC values for all scanners and receiver coils and pairwise comparisons. *Italicized* results represent the highest ICC-values for pairwise comparisons

	All scanners	Philips Achieva 1.5T – Philips Ingenia CX 3T	Philips Achieva 1.5T – Siemens MAGNETOM Aera 1.5T	Philips Ingenia CX 3T – Siemens MAGNETOM Vida 3T	Siemens MAGNETOM Aera 1.5T – Siemens MAGNETOM Vida 3T	Siemens MAGNETOM Vida 3T – all coils
Whole brain	0.867	0.987	0.763	0.869	*0.993*	0.999
Total white matter	0.868	*0.999*	0.749	0.986	0.813	0.999
Total grey matter	0.799	*0.964*	0.803	0.724	0.906	0.999
Cerebral cortex	0.784	*0.961*	0.802	0.699	0.888	0.999
Frontal lobe right	0.841	0.888	0.793	0.82	*0.991*	0.996
Frontal lobe left	0.836	0.915	0.805	0.798	*0.976*	0.995
Parietal lobe right	0.75	*0.933*	0.854	0.661	0.76	0.993
Parietal lobe left	0.753	*0.919*	0.878	0.684	0.735	0.995
Occipital lobe right	0.794	*0.965*	0.949	0.729	0.723	0.995
Occipital lobe left	0.813	0.961	*0.976*	0.762	0.635	0.986
Temporal lobe right	0.766	*0.995*	0.751	0.641	0.923	0.995
Temporal lobe left	0.486	0.466	0.663	0.366	*0.954*	0.997
Hippocampus right	0.85	0.961	0.766	0.843	*0.978*	0.991
Hippocampus left	0.838	0.93	0.747	0.848	*0.978*	0.986
Parahippocampal Gyrus right	0.819	0.781	0.737	0.775	*0.963*	0.981
Parahippocampal Gyrus left	0.725	0.887	0.662	0.703	*0.954*	0.973
Entorhinal Cortex right	0.779	0.919	0.708	0.74	*0.941*	0.97
Entorhinal Cortex left	0.751	*0.927*	0.658	0.729	0.882	0.979
Putamen right	0.984	0.973	0.979	*0.992*	0.991	0.994
Putamen left	0.987	0.985	*0.996*	0.988	0.993	0.995
Thalamus right	0.944	*0.986*	0.948	0.916	0.957	0.995
Thalamus left	0.965	*0.99*	0.961	0.954	0.974	0.995
Brainstem	0.971	0.985	0.935	0.985	*0.999*	0.997
Mesencephalon	0.953	*0.985*	0.915	0.979	0.929	0.995
Pons	0.983	0.991	0.964	0.989	*0.998*	0.999
Cerebellar cortex	0.795	*0.978*	0.781	0.71	0.927	0.998
Lateral ventricle right	0.999	*1*	*1*	0.999	*1*	1
Lateral ventricle left	0.999	0.999	0.999	0.998	*1*	1
Third ventricle	0.996	0.993	0.993	*1*	0.986	0.99
Fourth ventricle	0.986	0.97	0.984	0.994	*0.997*	0.99

In the analysis of all scanners, despite the statistical significant differences, most regions showed a good agreement (0.5 < ICC < 0.75) with exception of the left temporal lobe, where the agreement was only poor (ICC = 0.486). For the volume of the putamen, thalamus, the mesencephalon and the pons and all four ventricles the ICC showed excellent agreement (ICC > 0.9). The pairwise comparisons revealed the same tendency that the ICC values for the same manufacturer are higher than those between the same field strengths from different scanner manufacturers. Exceptions here were the total white matter, where the 3T-scanners showed higher agreement (ICC = 0.986) than the two Siemens scanners (ICC = 0.813), the parietal lobes, where the 1.5T scanners showed higher agreement (right ICC = 0.854; left ICC = 0.878) than the Siemens scanners (right ICC 0.76; left ICC = 0.735), the occipital lobes, where the results between the two Siemens scanners were the lowest (right ICC 0.723; left ICC = 0.635), the left temporal lobe, where the ICC values between the two 1.5T scanners were higher (ICC = 0.663) compared to those of the two Philips scanners (ICC = 0.466) and the putamen scanners of different manufacturers showed the highest ICC values (right ICC = 0.992 between 3T scanners; left: ICC = 0.996 between 1.5T scanners). The analysis of the different receiver coils from the Siemens MAGNETOM Vida 3T showed excellent agreement (ICC > 0.9).

Table 6 shows the results of the percentile allocation results from different MRI-scanners for the before mentioned regions.

**Table 6:** Overview of means and standard deviation for percentile allocation results for the different MRI-scanners and different receiver coils in case of Siemens MAGNETOM Vida 3T

	Philips Achieva 1.5T		Philips Ingenia CX 3T		Siemens MAGNETOM Aera 1.5T		Siemens MAGNETOM Vida 3T		Siemens MAGNETOM Vida 3T		Siemens MAGNETOM Vida 3T	
							20-channel-coil		32-channel-coil		64-channel-coil	
	Mean	Standard deviation	Mean	Standard deviation	Mean	Standard deviation	Mean	Standard deviation	Mean	Standard deviation	Mean	Standard deviation
Whole Brain	17.87	16.64	30.61	24.88	64.68	31.2	60.2	31.27	62.69	31.21	61.46	30.06
Total White Matter	38.93	29.52	41.02	29.73	80.21	23.36	33.39	25.1	38.2	27.86	35.06	25.66
Total Grey Matter	16.13	23.24	27.25	29.53	35.69	31.6	67.79	24.81	67.78	25.36	68.57	23.34
Cerebral Cortex	14.22	21.11	24.67	28.64	30.65	28.26	63	23.37	63.39	23.38	63.4	21.79
Frontal Lobe right	16.51	21.59	23.11	25.65	34.65	28.87	39.57	27.33	43.42	27.75	41.57	26.56
Frontal Lobe left	15.18	19.7	25.92	26.02	33.18	28.12	45.54	27.55	51.31	26.18	48.27	26.51
Parietal Lobe right	16.1	20.93	25.91	26.16	24.87	29.71	58.14	26.58	57.31	25.76	57.31	25.76
Parietal Lobe left	14.41	18.92	24.7	25.08	21.97	29.56	54.37	26.63	52.47	27.25	56.08	23.85
Occipital Lobe right	38.89	36	47.67	36.81	38.42	33.3	73.28	25.22	73.6	25.6	69.39	28.17
Occipital Lobe left	38.65	26.97	48.99	26.68	32.32	27.94	76.9	20.31	71.5	24.98	75.04	22.64
Temporal Lobe right	26.29	24.52	27.95	24.41	53.65	27.46	76.81	21.4	72.14	24.02	76.35	19.62
Temporal Lobe left	19.51	26.91	28.91	30.73	50.02	29.27	66.56	26.03	65.58	26.05	64.71	26.4
Hippocampus right	36.65	28.26	45.58	28.79	59.08	31.04	65.09	27.67	63.03	30.07	64	26.87
Hippocampus left	29.64	28.23	40.78	31.76	49.98	33.74	54.84	30.83	52.61	32.92	54.33	32.67
Parahippocampal Gyrus right	37.17	23.67	55.2	29.41	64.68	30.55	76.78	23.95	78.39	26.72	76.85	25.24
Parahippocampal Gyrus left	36.13	25.81	57.32	24.91	69.98	22.68	78.7	16.84	83.33	16.93	79.72	19.14
Entorhinal Cortex right	33.7	23.07	42.04	26.13	66.27	28.73	78.58	24.23	75.79	27.41	77.52	29.09
Entorhinal Cortex left	30.5	23.04	44.17	27.1	61.2	27.79	76.38	27.02	77.62	25.39	74.01	26.42
Putamen right	48.64	33.25	55.63	34.69	52.76	33.44	54.83	32.44	54.87	32.98	53.44	33.06
Putamen left	56.3	29.62	64.21	29.7	50.51	29.17	54.61	29.88	55.6	31.13	54.72	30.35
Thalamus right	36.72	26.32	43.83	24.33	40.59	24.78	55.73	24.93	57.56	27.02	56.18	25.91
Thalamus left	32.93	27.64	48.25	30.67	44.19	30.89	56.21	31	53.53	30.83	56.51	30.94
Brainstem	44.75	25.46	55.5	24.53	54.17	23.4	56.36	26.63	54.15	26.51	56.2	25.81
Mesencephalon	46.33	24.93	57.3	25.9	55.34	24.21	59.14	27.08	56.79	28.26	60.33	27.09
Pons	48.31	25.57	53.98	25.82	51.07	25.74	52.23	26.99	52.45	27.17	53.15	26.56
Cerebellar Cortex	39.66	29.49	48.82	28.87	65.28	25.31	82.65	16.07	81.07	16.79	81.39	16.55
Lateral Ventricle right	47.42	37.37	47.02	37.69	44.13	38	44.64	37.81	45.09	37.5	44.9	37.65
Lateral Ventricle left	55.56	32.94	54.72	33.18	52.47	33.79	53.17	33.72	53.39	33.49	53.37	33.54
Third Ventricle	57.32	35.13	54.73	35.94	54.53	36.88	67.22	51.59	54.2	36.89	52.99	36.6
Fourth Ventricle	53.88	26.29	48.34	28.09	48.87	28.85	47.51	29.34	46.7	29.36	46.18	28.73

Table 7 illustrates the results of the ICC for the analysis of the corresponding percentiles of the obtained volumetric results.

**Table 7:** ICC values for all scanners and receiver coils and pairwise comparisons. *Italicized* results represent the highest ICC-values for pairwise comparisons

	All scanners	Philips Achieva 1.5T – Philips Ingenia CX 3T	Philips Achieva 1.5T – Siemens MAGNETOM Aera 1.5T	Philips Ingenia CX 3T – Siemens MAGNETOM Vida 3T	Siemens MAGNETOM Aera 1.5T – Siemens MAGNETOM Vida 3T	Siemens MAGNETOM Vida 3T – all coils
Whole brain	0.494	0.775	0.263	0.547	*0.986*	0.994
Total white matter	0.604	*0.995*	0.386	0.985	0.304	0.984
Total grey matter	0.541	*0.886*	0.734	0.392	0.539	0.995
Cerebral cortex	0.528	*0.877*	0.757	0.384	0.511	0.992
Frontal lobe right	0.774	0.944	0.719	0.715	*0.973*	0.915
Frontal lobe left	0.685	0.861	0.706	0.624	*0.885*	0.972
Parietal lobe right	0.606	*0.862*	0.853	0.536	0.51	0.993
Parietal lobe left	0.644	0.857	*0.868*	0.591	0.542	0.98
Occipital lobe right	0.747	0.967	*0.993*	0.638	0.513	0.966
Occipital lobe left	0.64	0.896	*0.945*	0.649	0.287	0.961
Temporal lobe right	0.483	*0.98*	0.521	0.29	0.624	0.938
Temporal lobe left	0.588	*0.932*	0.561	0.459	0.817	0.992
Hippocampus right	0.82	0.939	0.732	0.812	*0.959*	0.984
Hippocampus left	0.854	0.901	0.741	0.898	*0.974*	0.981
Parahippocampal Gyrus right	0.658	0.733	0.618	0.662	*0.843*	0.975
Parahippocampal Gyrus left	0.496	0.7	0.458	0.464	*0.83*	0.931
Entorhinal Cortex right	0.53	0.689	0.4	0.495	*0.823*	0.944
Entorhinal Cortex left	0.616	*0.831*	0.549	0.584	0.733	0.966
Putamen right	0.935	0.965	0.923	0.908	*0.99*	0.995
Putamen left	0.94	0.956	0.966	0.916	*0.978*	0.987
Thalamus right	0.849	*0.947*	0.942	0.825	0.792	0.987
Thalamus left	0.769	0.574	0.584	*0.978*	0.911	0.991
Brainstem	0.94	0.893	0.903	*0.981*	0.978	0.989
Mesencephalon	0.92	0.9	0.904	*0.939*	0.932	0.977
Pons	0.983	0.972	0.984	*0.991*	0.984	0.996
Cerebellar cortex	0.602	*0.939*	0.65	0.423	0.662	0.968
Lateral ventricle right	0.998	*1*	0.996	0.998	0.999	1
Lateral ventricle left	0.998	*0.999*	0.995	*0.999*	*0.999*	1
Third ventricle	0.994	0.991	*0.995*	0.993	*0.995*	0.996
Fourth ventricle	0.975	0.956	0.972	0.993	*0.995*	0.993

The correlations show a high variance ranging from poor to excellent and were generally lower than for the raw volume measurements. Again, results for scanners of the same manufacturer were mostly higher than for the scanners of the same field strength with the before mentioned exceptions. Results for different head coils again showed excellent agreement for all analysed regions.

## Discussion

The objective of this study was to investigate the effect of MRI hardware on the results of a software-based automated volumetric analysis. Our findings revealed statistically significant differences in all observed brain regions between scanners of different manufacturers and field strength. However, the use of different receiver coils at the same scanner only demonstrated minimal statistical differences.

MRI-based volumetry is more and more considered as a helpful additional parameter in assessment of neurodegenerative diseases compromising disease diagnosis, follow up of disease progression and monitoring of potential treatment effects [1]. However sources of variance need to be considered, especially when planning longitudinal and/or multicentre studies. These compromise scanner-related factors such as manufacturer, field strength and use of different receiver coils which were examined in this study.

The results of this study stand in contrast with a work from Wittens et al., who showed an excellent inter-scanner reproducibility using a software tool named icobrain dm (icometrix) between a Philips Achieva 1.5T and Ingenia 3T scanner as we used in our study. Also, the absolute volume differences were not statistically significant in that work [10]. Our work showed significant and large differences in volumetry in all assessed regions and even larger differences concerning the percentiles. At least, one can say that most of the trends not passing significance in post-hoc comparison and that the highest ICC values were found between the scanners of the same manufacturer which partially supports the results from the before mentioned study. Other studies revealed a large discrepancy in the inter-scanner reproducibility of brain volumetry when using different processing software [8, 11] which is supported by our results even if in literature other scanner types and volumetry tools were used.

Reasons for such inhomogeneities in the results could possibly be slight differences in the magnetic field homogeneity [12] of the various manufacturers or also the different homogeneous excitation depending on the transmitter coils. These variabilities were shown to be higher in outer brain regions toward the brain edges and central regions were shown to have lower coefficients of variation [12]. This is supported by the results of the present work where highest statistical differences and lowest ICC results were found in the regions towards the brain edges and highest ICC values were found in the central brain regions, namely structures of the basal ganglia, the hippocampus and the thalamus. This is particularly important to note as these regions are helpful in distinguishing subtypes of neurodegenerative diseases of the dementia and movement disorder spectrum. No statistical differences were observed in the post-hoc analyses of the ventricular system. This can be explained by the higher differences of the signal intensities between cerebrospinal fluid and brain parenchyma compared to signal intensities between grey and white matter. This phenomenon also expounds the relatively high ICC values of the analysed brainstem regions where only brain parenchyma and cerebrospinal fluid need to be distinguished. Possible methods to increase reproducibility across vendors, especially addressing differentiation of grey and white matter, are parametric imaging methods such as T1-relaxation-time-mapping [13]. However, this work aimed to investigate the clinical consequences and not the physical reasons for the discrepancies. Since these differences already exist within a clinic, it is very likely that these variations also exist between the MRIs of different hospitals and centres and might be even stronger. This leads to the fact that a comparison of own images with external acquired images or a comparison of different external acquired sequences will only be possible to a limited extent. Particular attention must be paid when the clinical question is the progression of atrophy or evaluation of atrophy patterns, as a slight difference in the measured volume could be due to the use of a different device, in which the volumetry results of the corresponding area is generally lower. Only the volume of the ventricular system seems to be very robust against the used hardware and the concomitant protocol variations. For this reason, some studies suggest to establish the ventricular volume as a first parameter for age-related brain atrophy [14]. Software companies note down that the comparability of volumetry results is not given if the image data were acquired from different scanners and/or with varying sequence parameters. The present work shows how important it is to consider this note. This problem could perhaps be avoided by normalization where proportions of the total brain volume or total intracranial volume are compared instead of absolute volumes. This could possibly compensate for slight differences. However, the extent to which this leads to usable results would have to be evaluated in further studies [15]. Several automated or semi-automated volumetry programmes also provide a classification of the volumetry results on a percentile curve using a normal collective. These normative data might be influenced by the scanners on which the collective was examined. The software used in this work also delivers an allocation of the volumetry results on a percentile, corrected for age, gender and total intracranial volume. This might lead to some degree of normalization and it could be expected that this might compensate for hardware bias. The present work shows that the calculated percentiles do not yield to higher ICC values and a better comparability. The percentiles in this work even reinforce the influence of hardware instead of compensating it. The consequence is that either the range of normal values must be very large when including different scanner manufacturers and field strengths or that normal values can only be valid for a specific scanner.

The MRI systems used in this study are equipped with different receiver coils. This can also lead to differences in signal-to-noise ratio. To overcome this potential bias, all three available head coils at the Siemens MAGNETOM Vida 3T were used and compared. No significant differences between the 20-, 32- and 64-channel coils were found. Only the results from the Philips Achieva 1.5T cannot be put into context as no 8‑channel receiver coil was available for the other devices for potential comparison. Indirect conclusion from this experiment is a good intra-scanner reproducibility for the Siemens MAGNETOM Vida 3T. Also the reposition of the subject does not affect the intra-subject results in our study. This stands in line with a study in which subjects were examined twice in different MRI scanners also showing low intra-scanner variability [8]. Our subjects were healthy and compliant, so precise and reproducible positioning was possible in our study. However, in clinical practice inconsistent placing of subjects in the MR-scanner is common due to different factors. This can influence the accuracy of volume measurement [16].

There were some limitations to this study. First, the study sample size was quite small and only consisted of young and healthy participants up to 30 years. Thus it does not allow any sufficient conclusions about disease related variabilities due to a potentially great variability of brain atrophy and lesions. However, a dataset of 60 3D-T1w sequences was collected allowing an examination of intra-subject differences. When planning studies compromising brain volumetry, patient-related factors need to be taken in consideration. These factors are influenceable, potentially influenceable, such as patient positioning or not influenceable, such as age and gender [17]. The influenceable factors such as time of the day, hydration status and medication were kept constant throughout this study. All scans were performed within a time limit of four hours in a randomized manner.

Second, parameter harmonization was not optimal. Nevertheless, aim of the study was to compare sequences that are used in clinical setting. The parameters were taken from routine MRI protocols without further modification. Even if some studies show that adaptation of MRI parameters may improve the reliability of volumetric analysis [18], this would be at the expense of relevance for clinical routine but should be considered for multicentre studies were volumetric brain analysis is planned. Our chosen parameters resembled parameters of other MR-scanner comparison studies that included multiplanar reconstructable T1-sequences [8, 19]. Parameters within the manufacturer were better harmonized, probably explaining the elevated comparability of the volumetric results within the manufacturers in this study. Yet, a study showed that differences in brain volumetry persist despite harmonization of MRI protocols. This study however was performed on Multiple sclerosis patients treated with disease modifying drugs during an interval of up to three months [20] whereas in the present work, all healthy subjects were each examined within four hours. Also other protocol variations, such as acquisition acceleration need to be considered as they also potentially cause a systematic bias on volumetric measurement results [21].

Only one volumetric tool was used in this study. As no gold standard is defined, further investigation is needed to examine the impact of different scanners, manufacturers and field strengths to different semi- or fully automated software tools. Up to now, few studies tested and compared repeatability und reproducibility of other volumetric software tools [22]. Consequently, further investigation and comparisons of mdbrain and other volumetric tools is needed. Also the used version of the segmentation tool has to be taken into account when performing volumetric analysis as this might impact the results [23]. It is also important to note that there may be discrepancies in the definition of brain regions between different segmentation programs. It should be noted that the hippocampus can be divided into hippocampal subfields. In this context, a standard resolution of 1 × 1 × 1 mm^3^ may not be sufficient for volumetric analysis of internal structures [24]. The present study focuses exclusively on the analysis of the hippocampus in a broader context.

This study shows therefore that for (longitudinal) examination of an individual, it is highly relevant with what kind of scanner type, field strength and protocol it is examined. Thus for creation of volumetric databases it might either be important to include more examinations from different hardware or to perform harmonization by sequence parameter choice or postprocessing to eliminate a technical bias when defining normal values. Alternatively own databases for each scanner would be necessary.

## Conclusion

In conclusion, this study demonstrates that choice of scanner manufacturer and field strength significantly influences the results of AI-based brain volumetry. Choice of receiver coil only had subordinate impact on the volumetry results. Our results do not give any assumption about mdbrain performance and do not imply that one scanner is superior or inferior to another. Nevertheless, it must be noted that there is no reliable comparability for different devices. Particularly in case of patients with the question of atrophy progression, it must be considered that the volumetry results can only be reliable if patients have their follow-up examinations on the same device with the same scan parameters as it is stated by the companies. This aspect should be given greater consideration in clinical routine and included in the planning of (follow-up) examinations as well as for planning of multicentre and/or longitudinal studies with different MR-scanner systems.

## Abbreviations

ICC: Intraclass Correlation Coefficient
MRI: Magnetic Resonance Imaging
T: Tesla
